# Supplementary material for: Observation of a phase transition within the domain walls of ferromagnetic Co3Sn2S2
Source: Nat Commun. 2022 May 30;13:3000. doi: 10.1038/s41467-022-30460-y (PMC9151713; doi:10.1038/s41467-022-30460-y)
Supplement: Supplementary file 2 — Supplementary Information [file 41467_2022_30460_MOESM2_ESM.docx]

**Supplementary Information**

“**Observation of a phase transition**

**within the domain walls of ferromagnetic Co_3_Sn_2_S_2_**”

1. **DC Kerr ellipticity on traversing a domain wall**

The unmodulated, dc Kerr ellipticity was measured as a function of position transverse to the domain walls. Typically, three consecutive scans through a wall were recorded to improve signal to noise ratio (Fig. S1(a)). The results were then fitted to an error function whose amplitude is proportional to the $z$ component of the magnetization, $M_{z}$, within a domain. $\Phi_{dc}$ is therefore a measure of the equilibrium $M_{z}$ in zero applied field.

The temperature dependence of $\Phi_{dc}$ is shown in Fig. S1(b), with two fits (red curves) that span the low temperature spin wave regime and the high temperature critical regime. In the high temperature regime, the best fit was obtained with Curie temperature $T_{c}=175.2\pm0.2 K$ and critical exponent $\beta=0.33\pm0.03$, which is in good agreement with $\beta=0.326$ predicted by the 3D Ising model.


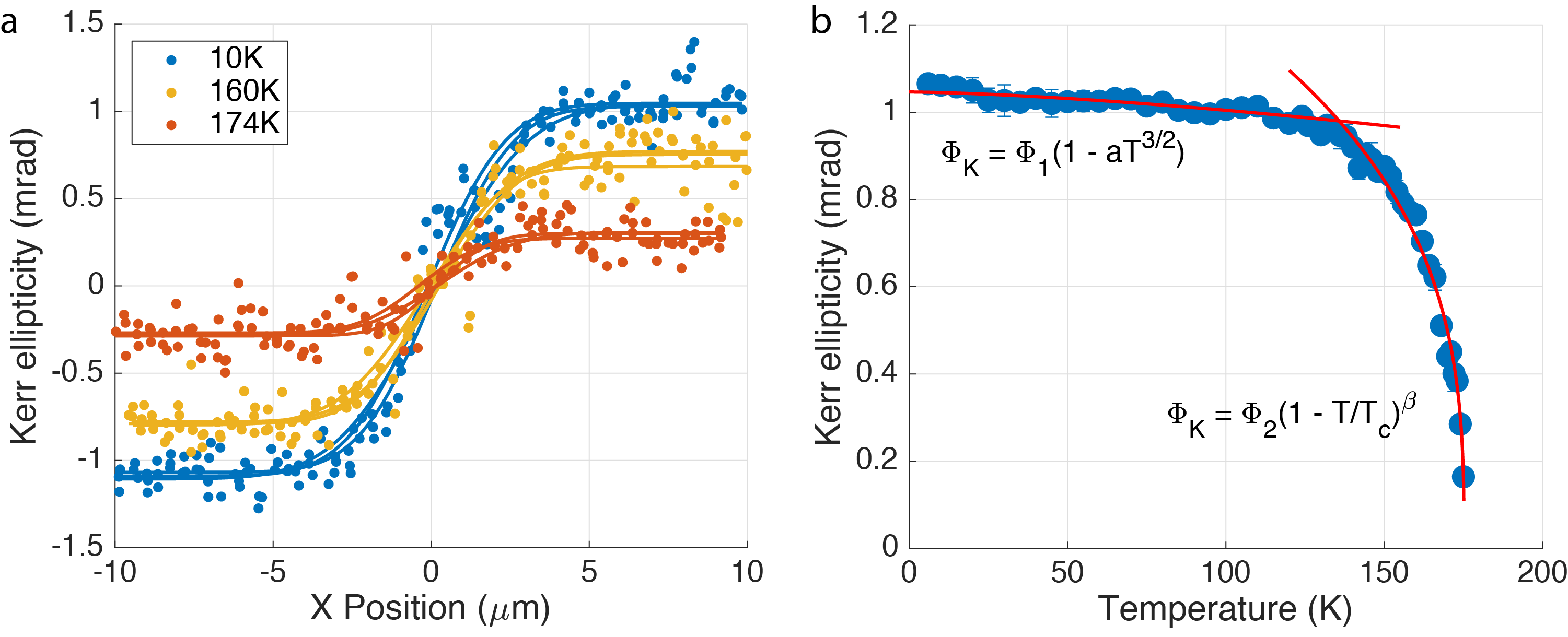


**Figure S1 | a** dc Kerr ellipticity plotted as a function of position relative to the center of the domain wall for various temperatures. The ellipticity data (dots) are subsequently fit to error functions (lines). **b** The temperature dependence of dc Kerr ellipticity compared to predictions for the spin wave (low temperature) and critical (high temperature) regimes, respectively.

1. **Comparison of unmodulated and ac Kerr ellipticity maps**

In order to verify that the ac Kerr response tracks the DW displacement, we show maps of unmodulated and ac Kerr ellipticity maps taken across the same region of the sample in Figs. S2(a) and (b), respectively.


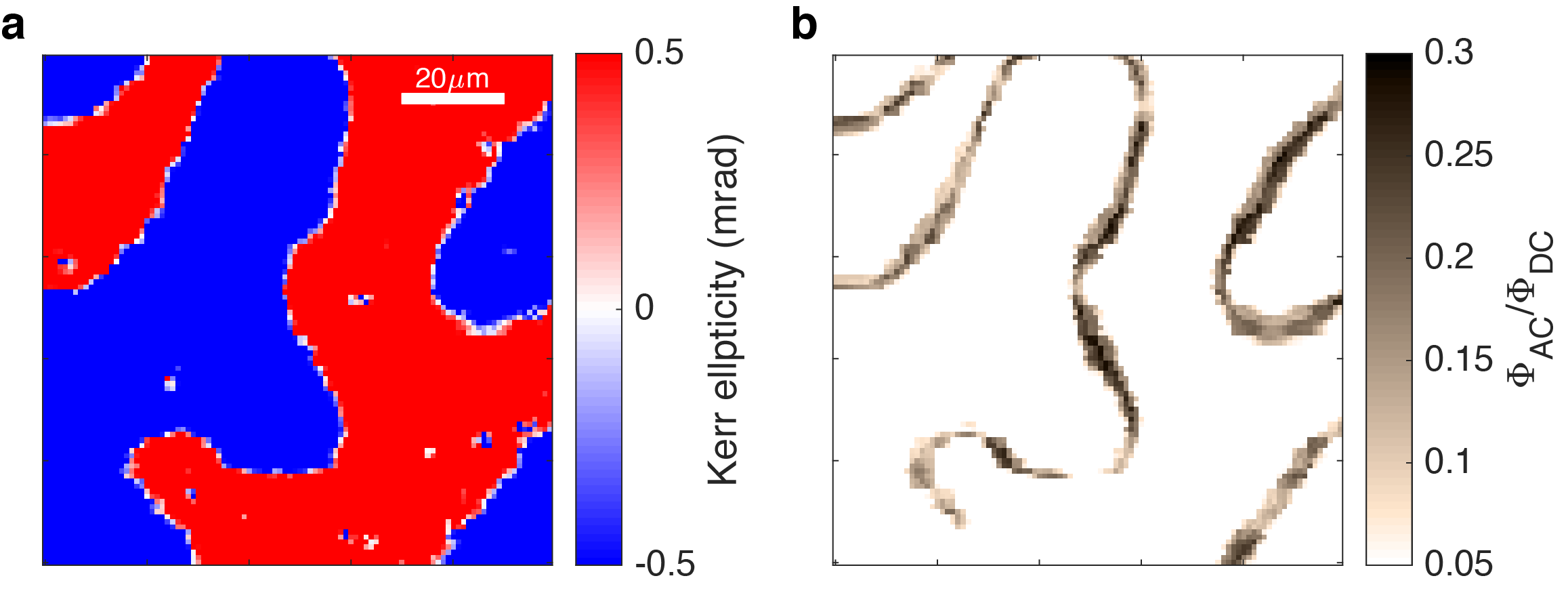


**Figure S2 | a** Unmodulated Kerr ellipticity map taken at T = 120 K reveals stripe-like magnetic domains. **b** Modulated (f = 1 kHz) ac Kerr map measured over the same region of sample exhibits a strong signal at the domain boundaries. The signal is normalized by the dc Kerr ellipticity obtained at 120 K.

1. **Analysis of domain wall displacement**

Domain wall (DW) displacement was determined by measuring both $\Phi_{dc}$ and the ac modulation synchronous with the applied magnetic field, $\delta\Phi_{ac}$, as a function of position along a direction normal to the DW. Scanning the laser probe transverse to the domain wall yields a peak in $\delta\Phi_{ac}/\Phi_{dc}$ at the DW center, as discussed in the main text. There are three length scales that determine the peak amplitude: DW displacement, $\Delta x$, DW width, $w$, and laser focus radius, $\sigma$. We obtain $\sigma\approx1 \mu m$ using standard optical methods for determining the focal diameter. We estimate that $w$ is on the order of several nanometers based on neutron scattering experiments, where a ferromagnetic spin wave dispersion was observed, $E\left( q \right)=E_{gap}+Dq^{2}$, with $E_{gap}\approx2 meV$ and $D\approx8 meV-nm^{2}$. Thus the our measurements are performed in the regime where $\sigma\gg w$, where the peak amplitude is independent of $w$ and related to the displacement through the formula, $\delta\Phi_{ac}(0)/\Phi_{dc}=erf \left( \Delta x/\sqrt{2}\sigma\right).$

1. **Spatial variation of the domain wall displacement**

The ac Kerr ellipticity exhibits a certain amount of spatial variation. Depending on the specific location of the measurement spot (Fig. S5a), the DW displacement can vary by a factor of two, while the threshold field $H_{th}$ exhibits a much smaller variation (Fig. S5b). However, the non-monotonic temperature dependence of the DW displacement is observed for all DWs, as shown in the ac maps plotted in Fig. 1 of the main text.

**
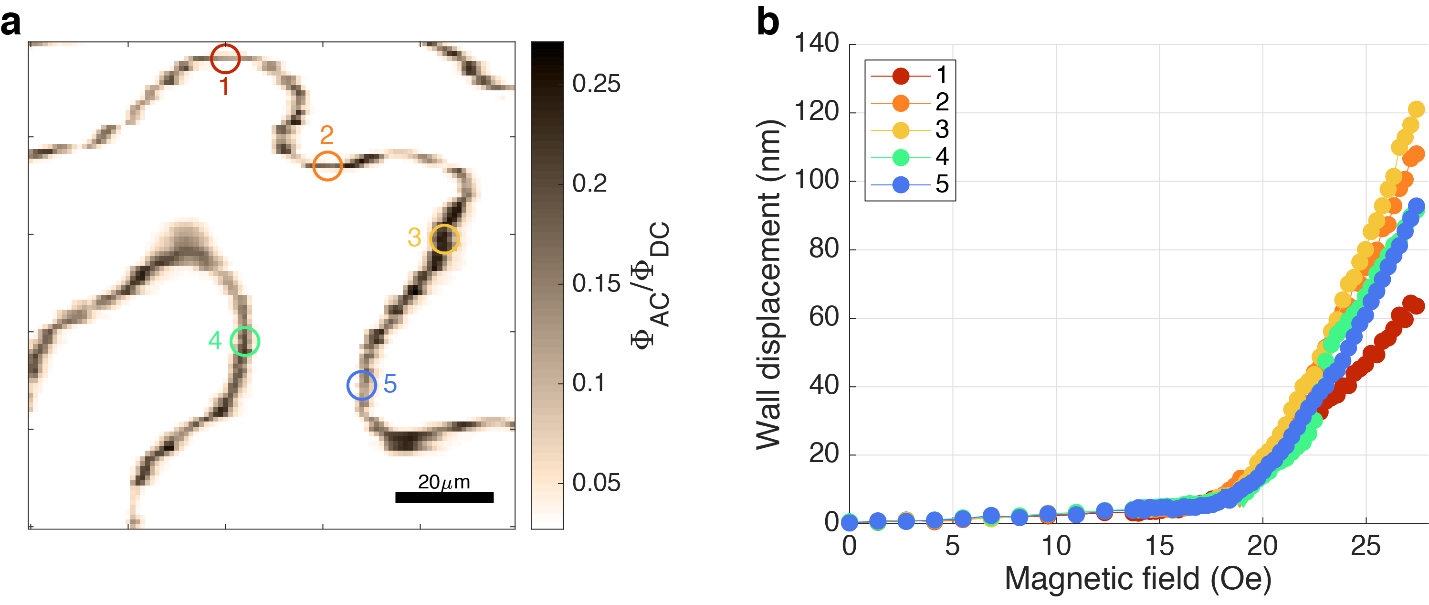
**

**Figure S3 | a** ac MOKE map measured at 130 K. **b** Plot of DW displacement at the five different locations indicated panel **a**, showing variations in DW mobility.

1. **Domain wall displacement at high temperatures (T > 140 K)**


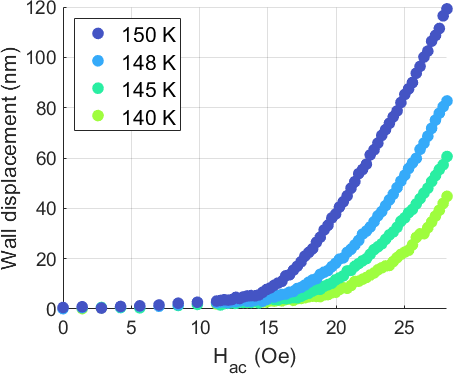


**Figure S4 | DW displacement above the BG phase transition.** Measurements of the domain wall displacement plotted against ac magnetic field amplitude at temperatures ranging from T=140 K to 150 K ($f=1 kHz$). Larger wall displacements and mobilities can be observed toward higher temperatures.

1. **Relation of domain wall mobility to Bulaevskii-Ginzburg order parameter**

We consider the propagation of an elliptical domain wall described by magnetization,

| $\boldsymbol{M}\left( x,t \right)=M{}_{s}\left[ \hat{\boldsymbol{z}}\tanh\left( \frac{x-vt}{w} \right)+\hat{\boldsymbol{y}}\frac{\rho}{\cosh\left( \frac{x-vt}{w} \right)} \right],$ | (1) |
| --- | --- |

where $M{}_{s}$ is the saturation magnetization, $w$ is the wall width, and $\rho=M_{y}/M_{s}$ is the normalized in-plane magnetization at the DW center. The power dissipated by the wall moving with velocity $v$ is given by,

| $P_{diss}={\frac{1}{\Gamma}}_{L}\int dx\dot{M^{2}}+\frac{1}{\Gamma_{\theta}}\int dx \dot{M_{y}^{2}},$ | (2) |
| --- | --- |

where $\Gamma_{L}$ and $\Gamma_{\theta}$ are relaxation rates of longitudinal and transverse fluctuations of magnetization.

Using change of variables $u=(x-vt)/w$,

| $P_{diss}=\frac{v^{2}}{w}\left[ \frac{1}{\Gamma_{L}}\int du\left( \frac{dM}{du} \right)^{2}+\frac{1}{\Gamma_{\theta}}\int du\left( \frac{dM_{y}}{du} \right)^{2} \right].$ | (3) |
| --- | --- |

The integral $\int du\left( \frac{dM}{du} \right)^{2}$was calculated numerically. For convenience in fitting we used the empirical form $(4/3)M_{s}^{2}\left( 1-\rho\right)^{2.2}$ which approximates the numerical result to within 2%. The integral $\int du\left( \frac{dM_{y}}{du} \right)^{2}$ gives $M_{s}^{2}\rho^{2}$, so that the dissipated power can be written,

| $P_{diss}=\frac{v^{2}M_{s}^{2}}{w}\left[ {\frac{1}{\Gamma}}_{L}\frac{2}{3}\left( 1-\rho\right)^{2.2}+\frac{\rho^{2}}{\Gamma_{\theta}} \right].$ | (4) |
| --- | --- |

For steady state motion we equate the dissipated power with the rate at which stored magnetostatic energy is lowered, $U=vM_{s}H$, where $H$ is the magnetic field applied parallel to the easy axis. Finally solving for the ratio of the velocity to the applied field yields the relation between mobility and BG order parameter plotted in Fig. 4(c) of the main text,

$$\mu=\frac{w}{M_{s}}\frac{1}{\frac{2}{3\Gamma_{L}}\left( 1-\rho\right)^{2.2}+\frac{\rho^{2}}{\Gamma_{\theta}}}.$$

1. **Various fits to the effective DW mobility**

**
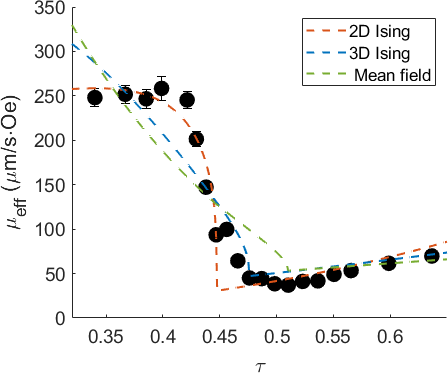
**

**Figure S5 | Effective DW mobility and fits to the data.** Effective mobility, $\mu_{\mathrm{eff}}=\frac{\Delta x}{\Delta H}$, of the DW is plotted against $\tau\equiv{4\chi}_{c}/\chi_{ab}$, which is the control parameter for the linear wall phase transition. The $\mu_{\mathrm{eff}}$ data fits much better with the 2D Ising model (red dotted line, $\beta=0.125$), compared to the 3D Ising model (blue dotted line, $\beta=0.326$) or the mean field theory (green dotted line, $\beta=0.5$) with critical values of $\tau$ near 0.5. In all cases, $\tau^{*}$ was adjusted give the best fit.

**References**

1 Garanin, D. A. Dynamics of elliptic domain walls. *Physica A: Statistical Mechanics and its Applications* **178**, 467-492, doi:10.1016/0378-4371(91)90033-9 (1991).
